# Raman spectroscopy: elucidation of biochemical changes in carcinogenesis of oesophagus

**DOI:** 10.1038/sj.bjc.6603102

**Published:** 2006-04-18

**Authors:** G Shetty, C Kendall, N Shepherd, N Stone, H Barr

**Affiliations:** 1 Biophotonics Research Group, Gloucestershire Royal Hospital, Great Western Road, Gloucester GL1 3NN, UK; 2 Department of Pathology, Gloucestershire Royal Hospital, Gloucester GL1 3NN, UK; 3 Department of Surgery, Gloucestershire Royal Hospital, Gloucester Gl1 3NN, UK

**Keywords:** Raman spectroscopy, adenocarcinoma, oesophagus, biochemical changes

## Abstract

Several techniques are under development to diagnose oesophageal adenocarcinoma at an earlier stage. We have demonstrated the potential of Raman spectroscopy, an optical diagnostic technique, for the identification and classification of malignant changes. However, there is no clear recognition of the biochemical changes that distinguish between the different stages of disease. Our aim is to understand these changes through Raman mapping studies. Raman spectral mapping was used to analyse 20-*μ*m sections of tissue from 29 snap-frozen oesophageal biopsies. Contiguous haematoxylin and eosin sections were reviewed by a consultant pathologist. Principal component analysis was used to identify the major differences between the spectra across each map. Pseudocolour score maps were generated and the peaks of corresponding loads identified enabling visualisation of the biochemical changes associated with malignancy. Changes were noted in the distribution of DNA, glycogen, lipids and proteins. The mean spectra obtained from selected regions demonstrate increased levels of glycogen in the squamous area compared with increased DNA levels in the abnormal region. Raman spectroscopy is a highly sensitive and specific technique for demonstration of biochemical changes in the carcinogenesis of Barrett's oesophagus. There is potential for *in vivo* application for real-time endoscopic optical diagnosis.

Despite advances in diagnosis and therapy, oesophageal adenocarcinoma remains an aggressive and usually lethal tumour (Zhang *et al*, 2005). Given the poor prognosis associated with the disease, a better understanding the pathogenesis and the factors associated with increased risk is essential. Also, strategies for prevention of oesophageal adenocarcinoma are hotly contested. The increased incidence of oesophageal adenocarcinoma is mirrored by similar rise in the incidence of Barrett's oesophagus (Prach *et al*, 1997). Barrett's oesophagus a premalignant condition is characterised by columnar mucosa, specifically intestinal metaplasia (IM) (Hamilton *et al*, 1988; Weinstein and Ippoliti, 1996).

Raman spectroscopy an optical diagnostic technique has been recognised as a valuable analytical tool in biological and medical research. It depends on an energy shift due to interaction with vibrational modes of molecules. The Raman spectrum is a direct function of the molecular composition of the tissue and provides a fingerprint of the tissue (Crow *et al*, 2003; Koljenovic *et al*, 2004; Stone *et al*, 2004).

During the past decade, progress in Raman spectroscopic instrumentation has increased the sensitivity of the measurement to such a degree that the acquisition of high-quality spectral data from biological tissue and cells is possible. The greatest benefit of this technique lies in its high sensitivity to subtle molecular and biochemical changes, as well as its capability for noninvasive sensing (Haka *et al*, 2002; Mantsch *et al*, 2002; Shafer-Peltier *et al*, 2002).

In previous studies, we have demonstrated the potential of Raman spectroscopy for the identification and classification of malignant changes in oesophagus. Raman spectroscopy was used to analyse 87 oesophageal biopsies from 44 patients. Spectral results were correlated with the consensus opinion of three gastrointestinal pathologists using multivariate statistical analysis (Kendall *et al*, 2003). However, there was no clear recognition of the biochemical changes that distinguished between the different stages of malignant progression. Our aim in this study was to understand these changes through Raman mapping and to attain direct biochemical information on Barrett's oesophagus, which is novel in this field.

This study utilises near-infrared Raman Spectroscopy, a highly specific optical analysis technique, to build robust spectral, biochemical diagnostic models of oesophageal pathologies. In doing so we will further our understanding of carcinogenesis in these tissues.

## MATERIALS AND METHODS

Ethical approval for this study was obtained from the Gloucestershire Local Research Ethics Committee. Informed consent was obtained from the patients at routine upper gastrointestinal endoscopy for surveillance of Barrett's oesophagus, chronic reflux symptoms and with suspected malignancy. Barrett's oesophagus was defined as the presence of columnar mucosa in the distal oesophagus of any length with IM on biopsy (Sharma *et al*, 1998). Patient demographics, including age and sex were noted.

In total, 29 samples collected from 22 patients were included in the study. The biopsies collected were snap-frozen in liquid nitrogen after mounting them on acetate paper. A consultant gastrointestinal pathologist who was blinded to identity and symptoms of the patient examined haematoxylin and eosin sections obtained from the frozen samples. The biopsy samples were classified as normal squamous, IM, low-grade dysplasia (LGD), high-grade dysplasia (HGD) and adenocarcinoma. Mixed samples with two pathologies were included in the study, which would mimic *in vivo* application. Owing to the focal change of mucosa often the scanned area will have heterogeneous pathology. A 20-*μ*m contiguous section for Raman mapping, mounted on calcium fluoride, was left uncontaminated thus providing an approximation to an *in vivo* approach.

### Raman spectral measurement

Raman spectra were measured with a highly sensitive Renishaw Raman System 1000® spectrometer optimised to provide rapid acquisition of high-quality tissue spectra. A diode laser with 830 nm incident light was used for excitation of the tissue. The spectrometer was connected to a microscope fitted with a Leica (NA 0.5) × 50 ultra long working distance objective lens and motorised stage to allow micro positioning of small tissue samples. A single 300 lines mm^−1^ grating was used to disperse the collected light and a charged coupled device detector measured the signal at each wavelength.

In total, 25 000 spectra were measured from the sections on calcium fluoride slides. The spectra were collected at 100-*μ*m steps across whole sections to provide information about global changes in the specimen. The contiguous haematoxylin and eosin section image captured by a digital camera mounted microscope was used to verify the different areas of dysplasia. The consultant pathologist compared these images with the white light images from the calcium fluoride section.

### Spectral analysis

Analysis was performed using Matlab and the PLS toolbox (Eigenvector Technologies, Manson, Washington, USA), generating principal components, which were used to identify the major differences between the spectra across each map. Pseudocolour maps of the principal component scores have been generated, and the peaks of the corresponding loads identified, enabling visualisation of the biochemical changes associated with malignancy (Pelletier, 2003). Reference spectra were obtained of glycogen, actin, triolein, DNA, collagen type 1, oleic acid (purchased from Sigma-Aldrich, Gillingham, UK).

Mean spectra from regions of interest were plotted for comparison and identification of spectral differences. Peak assignments of these mean spectra were also identified. Relative concentrations of different biochemical constituents were obtained by mathematical fit to mean spectra. This was performed as follows:

The PLS-toolbox® (Eigenvector) for Matlab® was adapted for use with the data in these studies. The fitting of the mean spectra of the biochemical constituents to the mean spectra of the different regions of interest from the tissue specimens was performed with ordinary least squares analysis. To optimise this process and remove any spectral offset the first derivative of each of the spectra was calculated using a Savitsky-Golay filter.

This fitting process is explained simply below. We begin by describing the measured tissue spectrum as a sum of the constituent spectra multiplied by their concentrations plus a residual or error. This leads to 
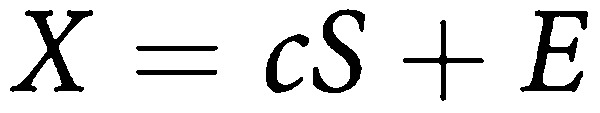
 where *S* is the matrix of spectral components, *c* is the matrix of concentrations to be predicted and *X* is the measured spectra. This can be used to provide a ‘best fit’ of the spectral components or basis spectra found within the measured spectra. The assumption is made that the residual E is minimised (i.e., least square of the residual) and that the spectral components selected are the major constituents of the spectra. 
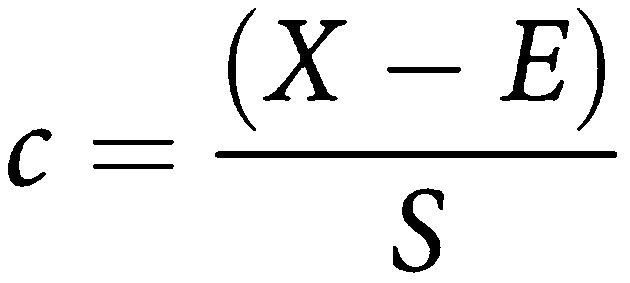


The disadvantage with this technique is that any colinearity in the components selected will skew the fit. An example being amino acids and the proteins containing them being used in the same model. Furthermore, if components are not included the omitted variable bias can introduce some errors in the fit. (Sowa *et al*, 2006) Observation of the residual *E* enables the quality of the fit to be observed and any remaining features of the spectra to be included in the next iteration of the model.

## RESULTS

In total, 29 samples with different pathologies were studied from 22 patients with male to female ratio of 26 : 4. The pathologies with normal squamous and adenocarcinoma and normal squamous and LGD constituted 24% each, normal squamous and HGD were 33%, normal squamous and IM were 14% and the rest were normal squamous and squamous dysplasia (Table 1).

**Figure 1:**
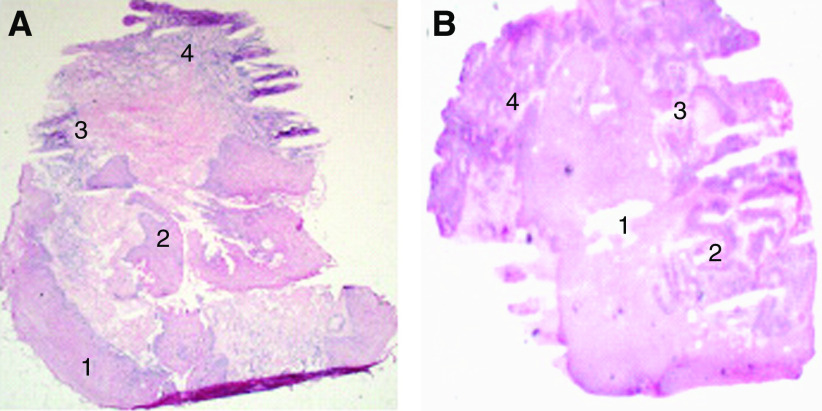
Haematoxylin and eosin stained sections with pathology labelled. (**A**) Regions 1 and 2 normal squamous and regions 3 and 4 HGD. (**B**) Region 1 normal squamous and regions 2, 3 and 4 adenocarcinoma.

**Figure 2:**
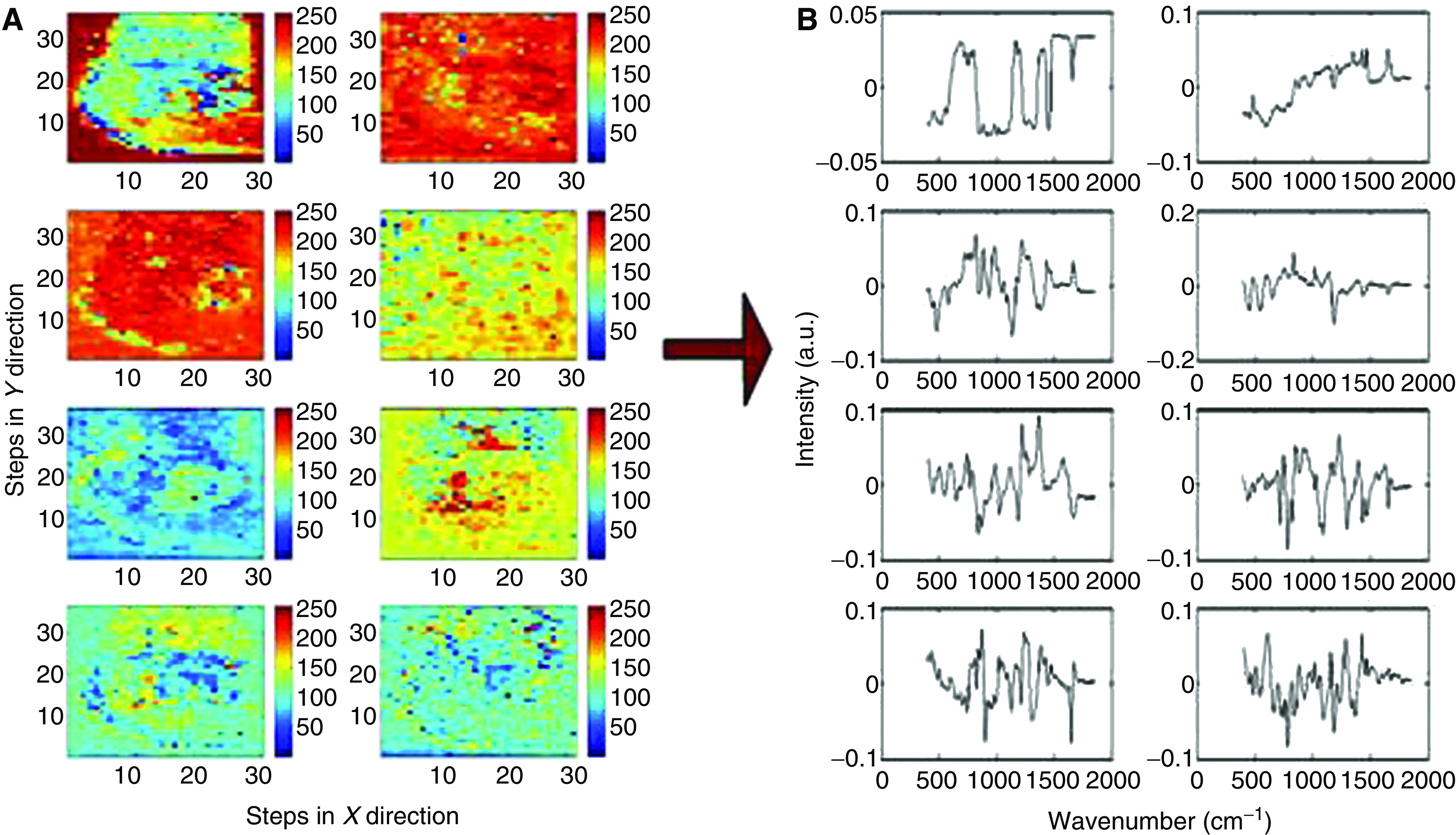
First eight pseudocolour principal component score maps and corresponding loads for the normal squamous and HGD sample (H&E stained section Figure 1A). PC 1 top left to PC 8 bottom right.

Figure 1 shows the haematoxylin and eosin section of the normal squamous and HGD (A) and normal squamous and adenocarcinoma (B) sections. The pseudocolour maps of PC scores and the corresponding loads (generated from the specimen during spectral analysis) of normal squamous and HGD illustrate the biochemical variation in the tissue. These are shown in Figure 2. The concentration distribution of different biochemical constituents in the various principal component score maps, vary from high-intensity colour red to low-intensity blue.

The first score map of normal squamous and HGD section shows the highest variation across the map between tissue and calcium fluoride slide. Peak assignment of the other following loads demonstrates further variation in the tissue distribution of DNA (deoxyribonucleic acid), glycogen, lipids and proteins. For example in load 3, DNA peaks can be demonstrated at 722, 752, 782 and 1663 cm^−1^ and peaks at 820, 884, 1223, 1278 and 1663 cm^−1^ correspond to proteins, including collagen I. The corresponding score shows variation in the DNA and proteins across the sample. Similarly, in load 6 peaks at 852 and 918 cm^−1^ correspond to glycogen and 750 and 918 cm^−1^ to lactic acid. Almost all inverted peaks in PC 6 load corresponds to DNA, which is concentrated in dysplastic area (Figure 1A). As the number of principal components increases more subtle variations can be seen. The variation in the lipids and DNA can be demonstrated across the sample in PC load 8 and its corresponding score.

**Figure 3:**
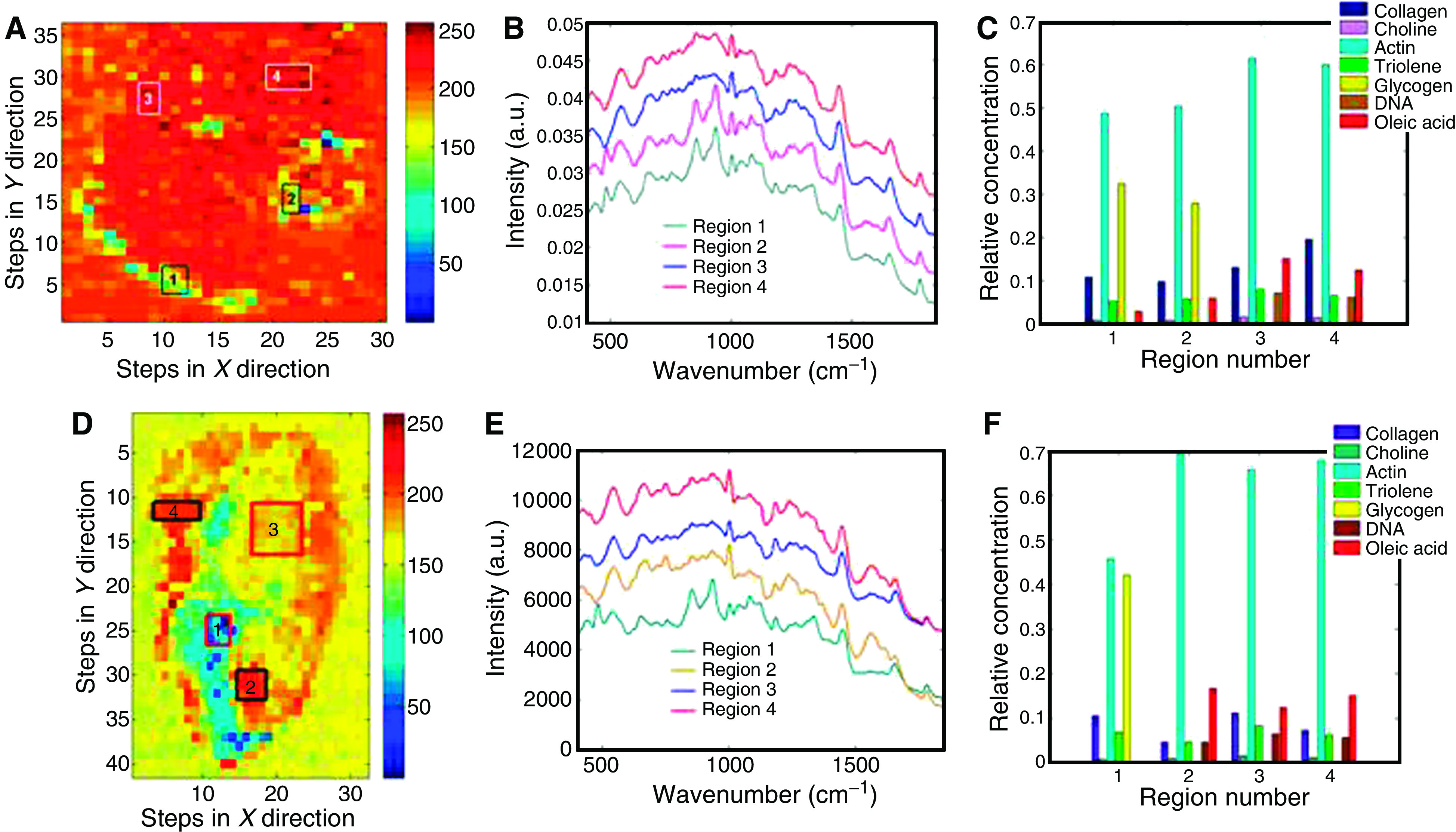
Normal squamous and HGD sample (H&E stained section Figure 1A). (**A**) Third principal component score map with selected regions marked. (**B**) Mean Raman spectra from selected regions. (**C**) Relative concentration of selected biochemical constituents calculated with mean spectra from selected regions. Normal squamous and adenocarcinoma sample (H&E stained section Figure 1B). (**D**) Third principal component score map with selected regions marked. (**E**) Mean Raman spectra from selected regions. (**F**) Relative concentration of selected biochemical constituents calculated with mean spectra from selected regions.

Figure 3 shows the score map from the third principal component (A) with the mean spectra from the selected four regions (B). Spectral differences were demonstrated between the selected regions, for example; glycogen peaks are seen at 853, 937 and 1333 cm^−1^ and DNA peaks can be clearly demonstrated at 719, 755 and 781 cm^−1^. The mean spectra from the selected regions were analysed for variation in the biochemical composition of the tissue (C). Following mathematical least squares fit, marked variation in the distribution of different biochemical constituents were seen; such as glycogen, DNA, oleic acid and collagen I across the tissue sample. DNA, oleic acid, collagen I and actin are predominantly seen in the region of HGD and glycogen in the normal squamous tissue.

An additional example of the distribution of various biochemical constituents is shown in Figure 3D–F. Figure 1B shows the section of normal squamous and adenocarcinoma with pathological distribution. The corresponding third principal component score shown in Figure 3D shows clear differentiation in the subpathologies, which has been delineated by colour contrast in pseudocolour map. The score map shows variation in the DNA and glycogen content across the sample. The mean spectra from the selected regions (1, 2, 3 and 4 in Figure 3D and F) were analysed to understand the biochemical changes associated in these regions. The analysis was carried out in a similar way to the previous sample and distinct variation was noted in the distribution of glycogen, DNA, actin and oleic acid. In Figure 3E the spectral difference for glycogen is more pronounced in region 1. The glycogen peaks at 481, 853, 937 and 1048 cm^−1^ in the mean spectrum of region 1 can be clearly demonstrated. On the contrary the DNA triplets at 720, 729 and 748 cm^−1^ are well seen in the spectra of regions 2, 3 and 4. The biochemical distribution plot shows increase distribution of DNA, actin and oleic acid in dysplastic glandular regions (2, 3 and 4 regions in Figure 3D–F).

## DISCUSSION

Better understanding of the carcinogenesis of Barrett's oesophagus is an essential step in targeting the lesion and improving the survival. Currently, endoscopy with biopsy during surveillance, or in suspected patients, is the gold standard for diagnosis of malignancy. In spite of various diagnostic methods and some surveillance programmes survival has not improved in last decade. The potential of present surveillance programmes to improve detection of adenocarcinoma at early stage has been questioned by many studies (Reid *et al*, 1988; van Sandick *et al*, 1998; Macdonald *et al*, 2000; Montgomery *et al*, 2001).

To improve mortality it is vital to diagnose the lesion at an early stage where there are only biochemical changes without significant architectural changes. A new diagnostic technique, which can facilitate this application, is valuable in future.

Various methods have been adopted to understand the molecular biology of cancer tissue. Most of these concentrate on DNA changes; however, little attention has been given to understand the other biochemical changes in the tissue (Jankowski *et al*, 1999; Doak *et al*, 2003). By understanding and quantifying the biochemical changes during neoplastic progression it may be possible to detect the abnormal lesion before morphological changes can occur.

For the development of new diagnostic methods for detection of tissue states *in vivo*, it is important to understand the spectral features of pure individual components of tissues; that means cells and subcellular components. Raman spectroscopy is still evolving at this stage.

Recent studies shows that Raman microspectroscopy has been applied to record complete spectral maps of freeze-dried cells as well as living cells in media without the need for any fixing technique before the mapping. The resulting spectra showed the localisation of the nucleus, organelles and the membrane (Kraft *et al*, 2003). Raman spectroscopy is more often associated with the determination of molecular structure and with qualitative analysis; as an example, the conformational characterisation of natively unfolded proteins through a simple band fitting of the amide I region has been recently described (Maiti *et al*, 2004). There have also been numerous applications of Raman spectroscopy for the quantitative analysis of sample composition, the most relevant application fields being clinical (Mc Laughlin *et al*, 2002; Pelletier, 2003). It needs further studies to unravel all the information or data present in the Raman spectrum.

Our results show Raman spectroscopy is a true indicator of biochemical changes in the tissue. The Raman effect is due to an energy shift due to interaction with molecular vibration modes, a Raman spectrum reflects the signature of that molecule. Overall we know the major changes in the cell such as increase in the DNA, however, delicate variation of different components (such as sugars, lipids, proteins and amino acids) needs attention. The subtle variations in the spectral peaks can confer ample biochemical information from the tissue.

The changes in the distribution of DNA, lipids and proteins in the cells during the process of carcinogenesis could be early markers in detection of high-risk patients before morphological changes occur. This study is a significant first step towards establishing these biochemical changes. By further understanding the carcinogenesis process it would help us to improve the diagnostic techniques, thus improving the survival. These preliminary results need further studies to confirm the results and to study more biochemical components. This will also need to be validated by different centres, and inter and intraobserver variability should be addressed.

Possible future applications of Raman spectroscopy include an *in vivo* endoscopic probe during surveillance, thus identifying high yield areas and eliminating the need for random biopsies. This may reduce sampling error and decrease or eliminate the need for biopsy that adds to the cost of the procedure. The Raman spectrum is a direct function of the molecular composition of the tissue and can therefore also be used to give a truly objective measure of pathology. Hence Raman spectroscopy can be potentially used as pathological tool in validating the diagnoses, identifying the margins in curative resection and lymph nodes.

## Figures and Tables

**Table 1:** Pathologies of different samples

**NSq and IM**	**NSq and LGD**	**NSq and HGD**	**NSq and adeno**	**NSq and SqDy**
4	7	10	7	1

Adeno, adenocarcinoma; HGD, high-grade dysplasia; IM, intestinal metaplasia; LGD, low-grade dysplasia; NSq, normal squamous; SqDy, squamous dysplasia.
